# Protecting older patients with cardiovascular diseases from COVID-19 complications using current medications

**DOI:** 10.1007/s41999-021-00504-5

**Published:** 2021-05-25

**Authors:** Mariana Alves, Marília Andreia Fernandes, Gülistan Bahat, Athanase Benetos, Hugo Clemente, Tomasz Grodzicki, Manuel Martínez-Sellés, Francesco Mattace-Raso, Chakravarthi Rajkumar, Andrea Ungar, Nikos Werner, Timo E. Strandberg, EuGMS Special Interest Group in Cardiovascular Medicine (Chairpersons A. Ungar and A. Benetos), Grodzicki, Strandberg

**Affiliations:** 1 Faculty of Medicine, Laboratory of Clinical Pharmacology and Therapeutics, Faculdade de Medicina, Serviço de Medicina III, Hospital Pulido Valente, CHULNUniversity of LisbonUniversidade de Lisboa, Lisbon, Portugal; 2 Department of Internal Medicine, Hospital Curry Cabral, Centro Hospitalar Universitário de Lisboa Central, Lisbon, Portugal; 3 Istanbul Medical School, Department of Internal Medicine, Division of Geriatrics, Istanbul University, Capa, 34093 Istanbul, Turkey; 4 Department of Geriatrics and FHU CARTAGE-PROFILES, CHRU de Nancy and INSERM 1116, Université de Lorraine, Vandoeuvre-lès-Nancy, France; 5 Department of Geriatrics, Centre Hospitalier de Wallonie Picarde, Tournai, Belgium; 6 Department of Internal Medicine and Gerontology, Jagiellonian University Medical College, Cracow, Poland; 7 Department of Cardiology, Hospital General Universitario Gregorio Marañón, CIBER-CV. Universidad Europea, Universidad Complutense, Madrid, Spain; 8 Division of Geriatrics, Department of Internal Medicine, Erasmus MC University Medical Center, Rotterdam, The Netherlands; 9 Brighton and Sussex Medical School, University of Sussex, Brighton, UK; 10 Department of Geriatrics and Intensive Care Unit, University of Florence and Azienda Ospedaliero Universitaria Careggi, Firenze, Italy; 11 Heart Center Trier, Krankenhaus der Barmherzigen Brüder, Trier, Germany; 12 Helsinki University and Helsinki University Hospital, Haartmaninkatu 4, PO Box 340, N00029 Helsinki, Finland; 13 University of Oulu, Center for Life Course Health Research, Oulu, Finland

**Keywords:** Antiarrhytmic, Anticoaculant, Aspirin, Colchicine, COVID-19, Diabetes, NSAID, RAAS, Statin, Vitamin D

## Abstract

**Purpose:**

In the pathogenesis of severe COVID-19 complications, derangements of renin–angiotensin–aldosterone system (RAAS), vascular endothelial dysfunction leading to inflammation and coagulopathy, and arrhythmias play an important role. Therefore, it is worth considering the use of currently available drugs to protect COVID-19 patients with cardiovascular diseases.

**Methods:**

We review the current experience of conventional cardiovascular drugs [angiotensin-converting enzyme (ACE) inhibitors, angiotensin receptor blockers, anticoagulants, acetosalicylic acid, antiarrhythmic drugs, statins] as well as some other drug classes (antidiabetic drugs, vitamin D and NSAIDs) frequently used by older patients with cardiovascular diseases. Data were sought from clinical databases for COVID-19 and appropriate key words. Conclusions and recommendations are based on a consensus among all authors.

**Results:**

Several cardiovascular drugs have a potential to protect patients with COVID-19, although evidence is largely based on retrospective, observational studies. Despite propensity score adjustments used in many analyses observational studies are not equivalent to randomised controlled trials (RCTs). Ongoing RCTs include treatment with antithrombotics, pulmonary vasodilators, RAAS-related drugs, and colchicine. RCTs in the acute phase of COVID-19 may not, however, recognise the benefits of long term anti-atherogenic therapies, such as statins.

**Conclusions:**

Most current cardiovascular drugs can be safely continued during COVID-19. Some drug classes may even be protective. Age-specific data are scarce, though, and conditions which are common in older patients (frailty, comorbidities, polypharmacy) must be individually considered for each drug group.

**Supplementary Information:**

The online version contains supplementary material available at 10.1007/s41999-021-00504-5.

## Key summary points

**Aim:**

To review current cardiovascular medications for benefits and potential harms during COVID-19.

**Findings:**

Several cardiovascular drugs have a potential to protect patients with COVID-19, although evidence is largely based on observational studies and age-specific data are scarce.

**Message:**

Most current cardiovascular drugs can be safely continued during COVID-19, but general conditions common in older patients must be considered.

**Supplementary Information:**

The online version contains supplementary material available at 10.1007/s41999-021-00504-5.

## Background

Ongoing SARS-CoV-2 (coronavirus) pandemic causing COVID-19 is potentially life threatening to older people with comorbidities. Males are more vulnerable than females, and patients with cardiometabolic diseases have higher risk for COVID-19-related severe complications and death. Complications are many, but cardiovascular and pulmonary ones are important and partly explain the susceptibility of patients with cardiovascular disease (CVD) to COVID-19 complications.

There is increasing knowledge of the potential benefits of steroids and specific antiviral treatments (e.g., remdesivir) in COVID-19 [1] and vaccination programs have commenced globally at end of 2020. However, the outcome of viral infection does not hinge only on the specific effects of the invading pathogen, but it also depends on the host response and premorbid condition. Immune response of the host may be inappropriate, either too strong causing collateral damage (cytokine storm), or too weak due to ageing and diseases. Differences in immune function (both innate and adaptive) between males and females may explain sex differences in susceptibility to complications. Besides the immune function, other factors in the host factors are also important. Any infection is potentially more dangerous to a patient with CVD as compared to healthy individual, especially because of “endotheliitis” [2] predisposing to thrombotic and thromboembolic complications.

Hence, it is worthwhile considering using currently available cardiovascular drugs to improve prognosis in COVID-19 patients with CVD. In the pathogenesis of severe COVID-19 complications, vascular endothelial dysfunction seems to play an important role as a root cause for coagulopathy, thrombosis, platelet activation and heightened inflammatory response [2]. Accordingly, risk of myocardial infarction, stroke, deep venous thrombosis and pulmonary embolism are increased, and geriatricians need to be aware of these complications. Because coronavirus uses angiotensin-converting enzyme 2 (ACE2) as its receptor, molecular mechanisms include derangements of renin–angiotensin–aldosterone system (RAAS) [3]. Several existing cardiovascular drugs and other drugs have beneficial effects on endothelial function/RAAS and on the sequalae of their dysfunction. This offers opportunities to improve prognosis by re-purposing these therapies.

At the moment evidence is collected from retrospective, observational studies, which despite propensity score adjustments used in many analyses are not equivalent to randomized controlled trials (RCTs). A recent analysis of trials registered on ClinicalTrials.gov revealed that only 7% (*n* = 114) of interventional clinical trials related to COVID-19 plan to evaluate cardiovascular therapies, most of them in a single center and enrolling < 1000 COVID-19 inpatients (88% RCTs). Most common drug classes were antithrombotics, pulmonary vasodilators, RAAS-related drugs, and colchicine [4].

Although evidence from RCTs is generally considered as most powerful, an important caveat related to cardiovascular drugs must be mentioned. An RCT performed in the acute phase of COVID-19 will not necessarily recognise potential benefits of chronic use of cardiovascular drugs which prevent atherosclerosis and preserve endothelial function long term, e.g., statins started before COVID-19 event. Performing an RCT in this setting is extremely difficult, even impossible in practice. The situation may be different for cardiovascular drugs (e.g., antiarrhythmics, anticoagulants, antithrombotics, colchicine) causing acute benefits by their effects on inflammation, thrombosis and coagulation.

In the following we shall review available evidence of cardiovascular drug treatment, mostly from observational studies, and their relationship—benefits or harms—to COVID-19. Besides conventional cardiovascular drugs we also include a review of nonsteroidal anti-inflammatory drugs (NSAIDs, including colchicine), as well as vitamin D, because observational studies have linked low serum 25-hydroxyvitamin D concentrations with increased CVD risk [5]. Geriatric aspects are discussed where feasible, but age-specific data are usually lacking.

Data were sought from clinical databases (PubMed, Embase, preprints in medXriv) using COVID-19 and specific key words for each drug class. Interpretations of data and recommendations were based on a consensus among all authors.

## Renin–angiotensin–aldosterone system (RAAS) inhibitors

ACE2 is a receptor present in many tissues throughout the human body, with a remarkable expression in the lungs type II pneumocytes, and it is important in the control of anti-atherosclerosis processes and blood pressure [6]. ACE2 is an aminopeptidase that converts Angiotensin (Ang) II into Ang [1–7]. Ang II exerts powerful vasoconstrictor, pro-fibrotic, and pro-inflammatory effects through AT1 receptors. In contrast, Ang [1–7] is a potent vasodilator, anti-apoptotic, and anti-proliferative agent through Mas receptors. SARS-CoV-2 uses the ACE2 as a cellular receptor to entry the host cells [7]. In particular, the spike protein of SARS-CoV-2 is processed by transmembrane protease-serine 2 (TMPRSS2) and favors the binding of the spike protein to ACE2 [8].

Angiotensin-converting enzyme inhibitors (ACEi) and angiotensin-receptor blockers (ARB) increase the ACE2 receptor [8]. This led to the hypothesis that these drugs could promote the inoculation of SARS-CoV-2 and potentially leading to a higher risk of infection and/or disease severity [9]. This hypothesis generated social concern among population and academics.

Due to the fragility of this harmful assumption many medical societies recommended not to withdraw ACEi and/or ARB due to their proved benefits in many diseases, such as hypertension, heart failure, coronary heart disease, diabetes mellitus, cerebrovascular disease and chronic kidney disease [10–12] and advocate an individualised treatment based on age, clinical condition, and co-morbidities of each patient, weighing the benefits of an effective treatment against the risk of abrupt drug discontinuation [13]. Indeed, subsequent systematic reviews and a recent RCT [14] support this recommendation [15, 16]. Moreover, the infusion of the soluble isoform of ACE2 is being studied as a decoy receptor for SARS-CoV-2 particles, reducing infectivity and conserving cardioprotective actions via Ang [1–8].

Focusing in hypertensive patients, pooled data showed a possible benefit on the severity of SARS-CoV-2 infection in patients on ACEi and/or ARB treatment [15–18]. Ssentongo et al. [18] reported a significant 35% lower risk of COVID-19 mortality (7 studies) among hypertensive hospitalised patients on ACEi/ARB.

Regarding SARS-CoV-2 infection no clear difference was found between ACEi and ARB [15]. The putative protective mechanism is still speculative, but hypothetically lung protection may be provided through the activation of angiotensin II-receptors type 2 and G protein coupled MAS receptors [19].

Thus, current evidence supports that ACEi and ARB are not harmful in COVID-19. The evidence to support a potential benefit in hypertensive subgroup is growing; however, it is still based on very low grade of certainty and the results of ongoing clinical trials are needed to clarify this hypothesis [20–22]. Due the broad and well-known benefits of these drugs in cardiovascular risk and blood pressure control, their prescription should anyhow be encouraged according to previously known clinical indications irrespective of age [23].

Other RAAS drugs such as renin inhibitors (aliskiren), mineralocorticoid receptor antagonists (spironolactone or eplerenone), or even sacubitril, a neprilysin inhibitor, are much less studied, but also much less prescribed [24]. Ongoing clinical trials [25] are needed to clarify this theoretical hypothesis of benefit regarding mineralocorticoid receptor antagonists, till there physicians should follow current recommendations for prescribing these drugs [26]. Sacubitril/valsartan association has also been a target for theoretical advantages mainly due to potential anti-inflammatory effect; however, evidence is still lacking [27, 28].

### In conclusion

ACEi and ARB have shown to be safe for COVID-19 patients and their prescription should be encouraged according to previously known clinical indications, also for older patients. Data on other RAAS agents and on geriatric population are scarce and based on low grade evidence.

## Statins

Before COVID-19 era in a randomized placebo-controlled trial of unselected acute respiratory distress syndrome (ARDS) patients [Hydroxymethylglutaryl-CoA Reductase Inhibition with Simvastatin in Acute Lung Injury to Reduce Pulmonary Dysfunction-2 (HARP-2) Study], simvastatin started acutely was safe but did not affect prognosis [29]. This and the potential ACE2 stimulating effect of statins initially raised concerns about their effects in SARS-CoV-2 infection. However, later subgroup analyses of HARP-2 trial suggested that ARDS patients with hyperinflammatory response (one third of all) did benefit from simvastatin treatment [30] raising some hopes for benefits also in COVID-19.

We now have cumulative evidence from at least 27 observational studies during COVID-19 pandemic ([31–57], Supplementary Table 1). In all but one study [41, 58] the mortality risk of patients using statins is unchanged or lower than that of nonusers of statins after adjustments. The largest analysis about COVID-19-related death is a nationwide analysis of people with diabetes in England [type 1 (T1DM), *n* = 264 390; type 2 (T2DM), *n* = 2,874 020] [31]. Statin treatment was associated with a significant 38% lower risk of death in type 2 diabetes, and nonsignificant 18% lower risk in type 1 diabetes. A meta-analysis of other serious complications (13 studies about death risk) has been recently published [59]. Patients in these studies have been usually upper middle-aged, average age being around 60 years. An analysis of frail Belgian nursing home residents (average age 86 years) suggested that statin intake was associated with milder clinical symptoms during COVID-19, although mortality decrease (49%) was not significant [60].

Several explanations are possible and biological and clinical plausibility of benefits and various pleiotropic mechanisms have been recently reviewed [61, 62]. In addition to their established anti-atherosclerotic, antithrombotic and anti-inflammatory effects, statins also have immunomodulatory effects which may reduce risk of cytokine storm. Furthermore, statins may prevent virus entry by lowering cholesterol content of membranes, and even have direct antiviral effects. The most probable explanation could be, however, that patients at risk of cardiovascular complications of COVID-19 are better protected when using a statin. Their vasculature is apparently more resilient because of atherosclerosis prevention. Statins are known to improve endothelial function, and many complications related to COVID-19 are due to endothelial dysfunction [2]. Outside COVID-19, men aged 80 and older using statins had similar prognosis as nonusers despite more frequent multimorbidity and higher mortality risk [63]. A very recent population-based study in Italy showed that statin treatment is beneficial also among oldest and frail individuals [64].

Because observational studies of statins have not been unequivocal, confirmation from RCTs has been called for [59]. However, if a potential benefit of statin treatment is due to long term, ongoing use, then trials randomising patients during acute COVID-19 will not identify this benefit. To prove long-term benefits would require RCTs which currently seem unrealistic to be executed.

### In conclusion

Observational studies have shown that ongoing statin treatment is neutral, or even beneficial, for patients with COVID-19. Average age of patients in these studies has been around 60 years, but totality of evidence of statins (vide supra) suggests that benefits are independent of age. Ongoing statin treatment should not be discontinued, but there is no evidence of benefit (nor harm) if statin is started during COVID-19. Possible interactions should be considered if COVID-19 is being treated with antiretroviral drugs.

## Acetylsalicylic acid (ASA, aspirin)

Acetylsalicylic acid (ASA) has well known antithrombotic effects, and is routine treatment at low dose in the prevention of CVD. ASA has also an anti-inflammatory effect by modulating the overproduction of pro-inflammatory cytokines and chemokines. Eventually, ASA has also antiviral activity against DNA and RNA viruses, including human coronaviruses [65, 66]. Therefore, the question arises whether this drug might present an efficient treatment in patients with COVID-19 and some recent studies have investigated this issue.

In a study including 314 COVID-19 patients, ASA was independently associated with decreased risk of mechanical ventilation, intensive care unit admission, and in-hospital mortality, whereas there were no differences in major bleedings between ASA users and non-users [67]. A large observational study which included hospitalised COVID-19 patients, reported a significantly lower incidence of in-hospital death in patients who received intermediate—compared to prophylactic-dose anticoagulation, and, separately, in patients who received in-hospital ASA compared to those who received no antiplatelet therapy [68].Conversely, a recent meta-analysis has investigated the possible association of ASA use in reducing mortality in patients with COVID-19 [69]. This meta-analysis included only 3 studies with a total of 1,054 patients, mortality rates were not statistically different between ASA users (22.6%) and non-users (18.3%) suggesting no protective effect of ASA in COVID-19 patients.

### In conclusion

Micro- and macrovascular thrombotic events are among the hallmarks of COVID-19. The optimal management strategy to prevent thrombosis in critically-ill patients, younger and older, with COVID-19 remains unknown. A dedicated RCT is needed to assess the possible protective role of ASA in COVID-19 patients.

## Anticoagulants

Hypercoagulopathy leading to thromboembolic events is common in older COVID-19 patients and critically ill COVID-19 patients have a hypercoagulable profile despite the therapeutic (curative, intermediate or full) anticoagulant doses given [70]. Two meta-analysis in hospitalised COVID-19 found an estimated pooled incidence of venous thromboembolism (VTE) of 17% and 21%, with higher rates with routine screening [71, 72]. VTE significantly increased the odds of mortality by as high as 74% [71]. Also, an elevated D-dimer level is a critical risk factor for the mortality of the SARS-CoV-2 infection [73]. Thus, several scientific societies provided recommendations about stratification of VTE risk in hospitalised COVID-19 patients and strongly advised prophylactic (low or preventive dose) antithrombotic treatment with low molecular weight heparin (LMWH), fondaparinux or unfractioned heparin, unless contraindicated. [74–76] Some of these recommendations consider the possibility of giving intermediate-dose LMWH in patients with multiple risk factors [72] and extended prophylaxis after discharge [74, 75]. While waiting for trial evidence concerning all COVID-19 patients from proper RCTs, a recent paper recommends switching all patients already being treated with oral anticoagulants (both antivitamin K and direct oral anticoagulants) to parenteral heparin at therapeutic levels to avoid the risk of over- or under treatment. [77].

Evidence about the effects of anticoagulation in COVID-19 patients is growing but still limited. A systematic review found a benefit of anticoagulants among hospitalised COVID-19 adults and suggested that therapeutic doses might be associated with better survival compared to prophylactic doses [78], while another systematic review showed a slight tendency towards a reduction in the mortality rate among mechanically ventilated patients with COVID-19 receiving therapeutic-dose anticoagulation [79]. However, only few of the included studies were of good quality and no information was provided regarding various age groups. A meta-analysis of hospitalised COVID-19 patients showed that anticoagulation in therapeutic dose was beneficial [80]. An analysis of 4389 hospitalised COVID-19 adults found that anticoagulation was associated with lower mortality and intubation, with a trend towards a higher benefit with therapeutic doses [81]. The mean age was only 65 years and was lower in patients without anticoagulation. Interestingly, in 26 autopsies, 11 had thromboembolic disease, not clinically suspected. Data in advanced ages are scarce. A small retrospective study in older COVID-19 patients with interstitial pneumonia suggested a benefit of direct oral anticoagulants [82].

Alternative potential benefits of heparin have been reported in patients with COVID-19, including direct SARS-CoV-2 antiviral activity and anti-inflammatory properties. [83, 84]. However, this is still an area of uncertainty and RCTs are ongoing to clarify the best antithrombotic strategies in COVID-19 [85]. The use of anticoagulants should be balanced against the risks of bleeding. Intracranial hemorrhage seems to be relatively uncommon among COVID-19 patients but is associated with a high mortality rate and is mainly seen in older patients [86].

### In conclusion

Anticoagulants, particularly when used in therapeutic doses, not just prophylactic dosing, seem to be beneficial in hospitalised patients with COVID-19. However, data are based on little high-quality evidence and are particularly scarce in advanced age population and in patients without anticoagulant treatment before admission. Consequently, optimal antithrombotic therapy in older patients with COVID-19 is yet to be determined.

## Beta-blockers

The use of beta-blockers in older patients with COVID-19 infection is not controversial, as studies published so far have suggested less risk associated with their use [87, 88]. At the same time, people with co-morbidities (heart failure, hypertension, diabetes, ischemic heart disease or atrial fibrillation) that are indications for the use of beta-blockers are at increased risk for severe complications, including death, during infection caused by the SARS-2 virus. These two observations indicate that there is no recommendation for withdrawal of beta-blockers during COVID-19.

Moreover, there are suggestions about the beneficial effects of beta-blocking agents, which would result from the potential role of beta-blockade in inhibiting the inflammatory response [89]. Several studies have indicated the role of catecholamines and stimulation of beta receptors in promoting the inflammatory response, as the overall effect of *β*2-adrenergic stimulation is an exacerbation of inflammation, promotion of B cell antibody production, and stimulation of dentritic cells and macrophages to secrete proinflammatory cytokines [89]. The multi-organ failure observed during cytokine storm can be partially linked to the activation of beta-receptors in many SARS-CoV-2 target organs such as lungs, heart, gastrointestinal tract, liver, vascular smooth muscle, and skeletal muscle. Moreover, *β*2-adrenergic receptors are expressed by the cells of the immune system, including macrophages, dentritic cells, and T and B lymphocytes.

The following hypothesised mechanisms of potentially beneficial effects of beta-blockade in different phases of COVID-19 have been proposed [90]:

- reduction of the SARS-CoV-2 host cell entry.
- decrease in proinflammatory cytokines production and inhibit cytokine storm.
- potential benefit in septic shock and ARDS.
- reduction of the hypercoagulation state.
- improvement of oxygenation level by beta-blockers.
- potential reduction of mucus hypersecretion.

### In conclusion

Taking into account the results of observational studies beta-blockers should not be stopped during COVID-19, and this applies also to older patients. Whether or not the anti-inflammatory effect of beta-blockers plays any clinically important role in prevention or treatment of cytokine storm has to be confirmed in prospective research and RCTs.

## Antiarrhythmic drugs (other than beta-blockers)

Arrhythmias are related to an increased morbidity and mortality in COVID-19, and their inpatient incidence is between 7.9% and 16.7%, possibly increasing up to 44% in patients admitted to intensive care unit [91]. Antiarrhythmic drugs diversely affect ion channels [92], of which particularly those mediating the selective passage of Ca^2+^ across cell membranes, modulate virus life cycles and play an important role in virus–host interaction [93–95]. Hence, bearing in mind the mechanisms of action and considering pleiotropic effects of some antiarrhythmic drugs, as well as the viral life cycle, it is natural to postulate they could work as antiviral drugs and affect SARS-CoV-2 infection.

In a large population study to assess the protective effect of several drugs acquired at the pharmacies < 30 days preceding diagnosis of severe COVID-19, flecainide was one of the drugs associated with significantly reduced risk of infection and hospitalisation [96]. On a bioinformatic level, propafenone revealed activity against the main protease of SARS-CoV-2 and influenced its virulence. Regardless these promising results, clinical studies are obligatory [97].

Amiodarone, used to control a wide range of atrial and ventricular arrhythmias, has been suggested as potential drug for prevention or treatment of COVID-19 [92, 98]. The ReCOVery-SIRIO, a multicenter study, aims to determine the role of amiodarone or verapamil, in hospitalised patients within the initial stage of COVID-19 [93, 95]. Dronedarone, a noniodinated and less lipophilic derivate developed to treat atrial fibrillation and flutter, has a better safety profile than amiodarone [92, 99].

Ca^2+^-channel blockers diltiazem and verapamil, applied in the prevention and treatment of supraventricular tachycardias [92], have been hypothesised to affect SARS-CoV-2 infection by inhibiting Ca^2+^ entry via L-type voltage-gated Ca^2+^ channels, with ensuing antiviral effects [93, 95].

Also digoxin, a cardiac glycoside used in the rate control of atrial fibrillation and flutter [92] has antiviral activity [100]. There is scarce research relating the remaining antiarrhythmic drugs, including ivabradine, a selective I(*f*) current inhibitor to COVID-19.

### In conclusion

Use of antiarrhythmic drugs in COVID-19 should be similar to patients in other diseases [98, 101]. Dose adjustments may be needed in older patients, and with renal or hepatic impairment [92, 102]. Despite intriguing experimental data and hypotheses of antiviral effects, these drugs are not indicated as a prophylactic therapy of SARS-CoV-2 infection at the moment.

## Antidiabetic drugs

Diabetes is one of the most important comorbidities related to the severity of SARS-CoV-2 infection [103] and often associated with CVD. Reported prevalence of diabetes among COVID-19 patients ranges from 5% to as high as 58%, depending on the patient and study criteria [104, 105].

Recommendations for diabetes treatment in COVID-19 advocate intensification of glycaemic control as a means of primary prevention for a severe illness and antidiabetic drugs should not be discontinued in the absence of specific contraindications [106]. Moreover, some antidiabetic drugs have shown pleiotropic activity added to the glycaemic control and may represent benefits to fight SARS-CoV-2 infection [103].Moreover, some antidiabetic drugs have shown pleiotropic activity added to the glycaemic control, particularly potential antiproliferative and immunomodulatory effects, and may represent benefits to fight SARS-CoV-2 infection [103].

### Metformin

Metformin was related to a decreased risk of mortality in patients with chronic lower respiratory diseases [104]. Regarding COVID-19, observational studies have suggested that metformin use in hospitalised patients with T2DM is associated with reduced mortality [107]. In the CORONADO (Coronavirus SARS-CoV-2 and Diabetes Outcomes) multicenter study (*n* = 1317), metformin use on admission (56.6%) was associated with a 41% reduced 7-day mortality risk [108]. A large US study showed that the reduction in mortality is gender-dependent: 24% for females and nonsignificant for males in propensity-matched model [109]. The associated benefits of metformin on mortality due to COVID-19 are not restricted to patients with severe disease or hospitalisation. A retrospective analysis in nursing homes demonstrated similar results, showing that residents taking metformin had a significantly reduced 30-day mortality from COVID-19 diagnosis compared to residents without antidiabetic drugs [110].

Regarding safety, metformin may induce lactic acidosis in cases of multi-organ dysfunction necessitating discontinuation of the drug [111, 112]. However, it can be safely continued in stable patients with normal oral intake and do not have nausea and vomiting. Dosing adjustment or interruption should be performed according to renal function [103, 113–115].

### Thiazolidinediones

Experimental studies suggest that pioglitazone may upregulate ACE2 in insulin-sensitive tissues and, although this effect in the lung is still uncertain, this supposed increase in ACE2 expression has raised some concerns about the use of this drug in patients with diabetes and COVID-19 [104, 115–117]. Moreover, thiazolidinediones seem to increase the risk of pneumonia when compared to sulfonylureas and, taking into consideration the risk of fluid retention, it could be judicious to withhold PPAR-γ agonists in acutely ill patients [104, 115, 116].

### Dipeptidyl peptidase-4 inhibitors (DPP-4i)

There is no sustained evidence to determine the impact of DPP-4i on clinical outcomes related to COVID-19 [103, 104, 107, 108, 114, 118–120]. As DPP-4i exhibit an optimal safety profile, even in fragile people, they remain a valid therapeutic option for the management of patients with T2DM and COVID-19 who are stable and with satisfactory oral intake [115, 120]. However, dose adjustments may be required according to renal function and more data are needed for recommendations in critically ill patients [114, 115].

### Glucagon-like peptide-1 receptor agonists (GLP-1 RAs)

GLP-1 RAs have shown to be particularly effective in obese T2DM patients and are indicated for patients at high risk or established atherosclerotic CVD due to evidence of cardiovascular protective effects [113, 121]. However, none of the studies have demonstrated clear influence on outcomes related to COVID-19, and there is lack of evidence regarding the safety of use of GLP-1 RA in critically ill patients. Their use should be carefully considered or discontinued in the most fragile patients [114, 115, 121].

### Sodium-glucose co-transporter-2 inhibitors (SGLT2is)

SGLT2is are highly effective in the treatment of diabetes and have been strongly associated with improved CVD outcomes [103, 113, 114]. A multicenter RCT ((Dapagliflozin in Respiratory Failure in Patients with COVID-19—DARE-19) is underway to investigate the role of dapagliflozin in reducing disease progression, complications and all-cause mortality in COVID-19 [122].

SGLT2is are associated with an increased risk of volume depletion and euglycemic ketoacidosis, which could be greater in the context of severe infections. Therefore, it is recommended to withhold them in hospitalised patients with COVID-19, and also in very unwell outpatients [106, 109, 113, 114, 116, 120, 122].

### Sulfonylureas

The number of patients using sulfonylureas has been low in observational studies of COVID-19, probably due to adverse effects [107, 122]. Although sulfonylureas and glinides apparently do not directly affect SARS-CoV-2, it is recommended to withhold them in patients with compromised oral intake or severe illness, to avoid the risk of hypoglycemia [114–116, 123].

### Insulin

Insulin is a safe choice under most circumstances, being the only therapy for people with T1DM and considered the first-line treatment in hyperglycaemic critically ill patients [113, 117].

Several studies have shown that insulin therapy was linked to poor prognosis in COVID-19 [107, 108, 110]. However, it is important to underline that the poorer outcomes are probably due to the fact that use of insulin reflects more advanced diabetes and patients who are often older and frailer. Indeed, insulin often remains the only drug to achieve glucose control in critically ill patients, particularly those in intensive care units, where other glucose lowering drugs must be discontinued [105]. Moreover, given the wide clinical spectrum of COVID-19 and potential to deteriorate rapidly, it is reasonable to consider early introduction of insulin on hospital admission [116]. Hypoglycaemia should be avoided as it is also related to unfavourable outcomes [114, 115].

### In conclusion

Data linking diabetes and COVID-19 are growing but still limited, especially in older population. An optimal glycaemic control is crucial to improve prognosis in COVID-19 patients with diabetes and pharmacological treatment must be carefully managed to avoid serious adverse reactions, particularly in the most fragile patients. There is evidence of extrapancreatic pleiotropic effects for some antidiabetic drugs that can represent a potential benefit in the context of COVID-19, but these effects specifically in older patients are unknown.

## Vitamin D

Epidemiologic data suggest that the risk of infectious diseases is higher when 25-hydroxyvitamin D [25(OH)D] levels are < 20 ng/mL (50 nmol/L) and risk decreases with higher concentrations [124]. In RCTs vitamin D supplementation of 10–25 µg (400–1000units) a day had a small protective effect against acute respiratory infections [125, 126]. COVID-19 severity and mortality rates are higher in older adults, African Americans, patients with diabetes, chronic lung disease, and CVD—all groups with low vitamin D levels. Ecological studies suggest that high latitudes and winter season—risk factors for low vitamin D—are associated with higher mortality rates in COVID-19 [125, 126]. Consequently, the fact that risk for vitamin D deficiency overlaps with factors associated with mortality from COVID-19 has stimulated studies on the role of vitamin D in COVID-19, especially from a therapy viewpoint.

Because epidemiologic associations may be explained by the “healthy user” effect [127] clinical studies are needed. These have revealed mixed findings between serum 25(OH)D levels and risk for COVID-19 [128], and clear support for causality remains undetermined [126].

The subsequent question is whether vitamin D replacement or treatment with higher pharmacological doses may have a beneficial effect on COVID-19 risk, and/or severity and mortality. There is a strong rationale to study these effects [127]. The ability of adequate vitamin D status to prevent mortality associated with COVID-19 has been suggested by an increasing number of observational studies [129–131]. An example is the recent, large analysis from UK Biobank (*n* = 8297), where habitual use of vitamin D supplements was significantly associated with an adjusted 34% lower risk of COVID-19 [132]. Yet, there are also conflicting findings [124, 127, 130]. Regarding effect in treatment of COVID-19, a one small (*n* = 76), low quality RCT reported significantly reduced disease severity among patients given a high dose of vitamin D on admission [133]. Another small trial (*n* = 40) reported that patients with mild or asymptomatic COVID-19 and given high-dose vitamin D supplementation [1500 µg (60,000 units) daily for 7 days] more likely were negative at third week [134]. On the other hand, a single oral dose of 5000 µg (200,000 units) of vitamin D3 did not influence length of stay among patients with severe COVID-19 [135].

Currently, several larger, placebo-controlled trials with vitamin D are in progress [124, 127, 136, 137].

It is suggested that the effect of vitamin D is prominent when there is either extreme deficiency, or high therapeutic doses are used. Generally, vitamin D is safe, especially if the dose does not exceed the upper tolerable limit [129]. Suggested dosing of daily vitamin D varies: 15–25 µg (600–1000 units, 124), 25–50 µg (1000–2000 units, 129), or at daily equivalent doses of 25–100 µg (1000–4000 units), daily or weekly [127]. In general, daily dosing is recommended—rather than higher doses weekly or monthly [124, 129]—and mega-doses are not recommended. As target level in serum, some authors suggest a level > 20 ng/mL (50 nmol/L) [129]*,* many others set the target at 30 ng/mL (75 nmol/L)[127]. Nevertheless, one should consider that the beneficial effect is expected to be small and may take some time (months) to develop [129]. These conclusions may change by the newly emerging data.

Of note, the UK National Institute for Health and Care Excellence (NICE) COVID-19 rapid guideline: vitamin D (https://www.nice.org.uk/guidance/ng187) has been published very recently, indicating the validity of their existing advice that adults in the UK should take 10 µg (400 units) of vitamin D daily between October and March, while certain populations at risk of vitamin D deficiency should consider taking vitamin D daily throughout the year.

### In conclusion

The clinicians should consider the possibility of lowering the impact of COVID-19 by ensuring adequate vitamin D replacement in populations, where vitamin D deficiency is prevalent. These often include older adults with CVD. Although the evidence of benefit in COVID-19 is largely observational, adequate vitamin D intake is nevertheless recommended for older persons to preserve bone health [138].

## Non-steroidal anti-inflammatory drugs (NSAIDs) including colchicine

NSAIDs are widely used also by patients with CVD for pain, antipyretic effect, and management of inflammatory diseases. However, there have been concerns about the safety of NSAIDs use during COVID-19. It was speculated that ibuprofen can be detrimental when taken to treat general symptoms of a SARS-CoV-2 infection [139] However, on March 2020, the WHO released a statement after a literature review, that there is no evidence against the use of ibuprofen in COVID-19 patients [140] which was followed by confirmatory statements of FDA, EMEA, and others. These statements supported the continued use of NSAIDs for analgesia/antipyretic treatment and recommended continuation of regular therapy. The use of NSAIDs, such as ibuprofen, was not associated with severe COVID-19 or increased mortality in a Danish cohort study of 9236 patients [141]. Of them 248 (2.7%) had presumably taken an NSAID in the 30 days before being positive diagnosed for SARS-CoV-2. There were no significant differences between NSAID users and non-users for 30-day mortality (6.3% of vs. 6.1%), hospitalisation (24.5% vs. 21.2%), or intensive care unit admission (4.9% vs. 4.7%) [141]. An additional systematic review of NSAIDs in various respiratory infections including two RCTs gave inconclusive results mainly due to low quality of studies [142].

On the other hand, anti-viral and anti-inflammatory effects of NSAIDs could also be theoretically beneficial in COVID-19. Several in vitro and in vivo studies have evaluated NSAIDs in viral infections. The proposed mechanism of action, e.g., by indomethacin, is inhibition of intracellular viral RNA replication [143] and interference with virus-mediated signal transduction pathways and transcription factors. As a clinical example, indomethacin (25–50 mg of twice daily) treatment in > 60 non-hospitalised patients with suspected and confirmed COVID-19 was associated with symptomatic relief of incessant coughing and COVID-19-associated general symptoms [144]. Besides anti-viral properties, NSAIDs may have anti-inflammatory properties, e.g., by attenuating cytokine storm in ARDS. However, data are limited and clinical evidence is lacking so far.

A highly intriguing new observation is the potential role of colchicine, an old anti-inflammatory drug used for gout and pericarditis, in COVID-19. Recent RCTs in patients with coronary artery disease have shown significant reduction of CVD events with colchicine treatment [145]. A meta-analysis of eight studies included 5778 COVID‐19 patients and 2668 patients who received colchicine treatment [146]. Of the eight studies, three were RCTs, two and two retrospective and prospective cohort studies, respectively, and the remaining one was a case‐control study. In the pooled analysis colchicine was associated with improvement in outcomes of COVID‐19 by 57%, and a reduction of mortality also by 57%. The reduction of outcomes was somewhat smaller (49%) in RCTs than in observational studies (59%), but still significant [146]. In January 2021, the Montreal Heart Institute announced that in the large, international COLCORONA clinical trial with non-hospitalised patients, colchicine, as compared to placebo, has reduced hospitalisations significantly by 25%, the need for mechanical ventilation or deaths nonsignificantly by 50% and 44%, respectively, among 4159 patients in whom the diagnosis of COVID-19 was proven by a naso-pharyngeal PCR test [147]. At the moment the data for COLCORONA are only available as a preprint.

### In conclusion

Although there is no safety signal for detrimental effects of NSAID use in COVID-19, NSAID use need to be carefully evaluated in patients with CVD due to its association with kidney injury, and gastrointestinal complications, and increased risk of bleeding. This is especially important in older patients with COVID-19. Benefits and risks need to be individually evaluated. Currently, there is no evidence for initiation of NSAIDs as a specific therapeutic approach in COVID-19 patients. However, results from the meta-analysis and COLCORONA trial are apt to change this conclusion for colchicine.

## Overall conclusion

Summary of the benefits and potential harms of various drug classes and recommendations for their use in COVID-19 is presented in Table 1. While these recommendations are independent of patients’ age, drug-specific cautions due to frailty, renal or hepatic insufficiency, and potential comorbidities, contraindications, and drug interactions should be individually considered for all drug groups.

**Table 1:** Summary of recommendations for the use of currently available drugs to protect COVID-19 patients with cardiovascular diseases

Drug group	Benefits	Potential harms	Recommendation
Renin–angiotensin–aldosterone system (RAAS) inhibitors	May reduce risk of serious complications related to COVID-19 (observational evidence)	General to this drug group, especially electrolyte disturbances	Do not stop during COVID-19. Start if indicated for cardiovascular indications (hypertension, heart failure)
Statins	May reduce risk of serious complications related to COVID-19 (observational evidence)	General to this drug group, including muscle and renal adverse effects	Do not stop during COVID-19. Start if indicated for dyslipidemia indications
Anticoagulants	May reduce risk of serious complications related to COVID-19 (observational evidence)	General to this drug group, especially bleeding	Continue or start during COVID-19 to prevent thrombotic complications
Acetylsalicylic acid	May reduce risk of serious complications related to COVID-19 (observational evidence)	General to this drug group, especially gastrointestinal adverse effects	Continue or start during COVID-19 to prevent thrombotic complications
Beta-blockers	May reduce risk of serious complications related to COVID-19 (experimental data)	General to this drug group, especially bradycardia	Continue or start if indicated for cardiovascular indications
Antiarrhythmic drugs	May reduce risk of serious complications related to COVID-19	Various, depending on specific drug	Start if indicated for arrhythmias
Antidiabetic drugs	Optimal glycemic control is important during COVID-19	General to this drug group in older patients, most importantly hypoglycemia with	Do not stop without specific reasons during COVID-19. Start if indicated for hyperglycemic indications
Vitamin D	In deficiency, may reduce risk of serious complications related to COVID-19	Hypervitaminosis D leading to hypercalcemia	Do not stop during COVID-19. Start if deficiency of vitamin D
Non-steroidal anti-inflammatory drugs (NSAIDs)	Symptomatic treatment for pain and fever in COVID-19	General to this drug group in older patients, especially gastrointestinal and cardiovascular adverse effects	Start if indicated for pain/fever
Colchicine	May reduce risk of serious complications related to COVID-19 (promising observational and randomised controlled trial evidence)	General to this drug, especially gastrointestinal disorders	Start during COVID-19 (preliminary evidence)

There is insufficient data to make these recommendations specific for older patients, and individual characteristics such as frailty, renal and hepatic dysfunction, comorbidities, and potential drug interactions must be considered for each drug group

## Supplementary Information

Below is the link to the electronic supplementary material.

Supplementary file1 (DOCX 16 KB)
