# Supplementary material for: Protecting older patients with cardiovascular diseases from COVID-19 complications using current medications
Source: Eur Geriatr Med. 2021 May 25;12(4):725–39. doi: 10.1007/s41999-021-00504-5 (PMC8143992; doi:10.1007/s41999-021-00504-5)
Supplement: Supplementary file 1 — Supplementary file1 (DOCX 16 KB) [file 41999_2021_504_MOESM1_ESM.docx]

**Supplementary table**

Table 1. Observational studies of statin treatment and mortality during COVID-19

| Study  (references 31-57) | Country | No. patients | Average age, year | Statin treatment-associated mortality risk (adjusted and in-hospital unless otherwise indicated) during COVID-19; odds ratio or hazard ratio (95% confidence interval) |
| --- | --- | --- | --- | --- |
| Holman N et al | UK, national | type 2 diabetes,  2,874 020 | 70 | Risk of death due to COVID-19:  0.72 (0.69–0.75) |
| Mallow et al | USA, multicenter | 21 676 | 65 | 0.54 (0.49–0.60) |
| Lee et al | Korea, national | 10448 | 45 | 0.64 (0.43–0.95) |
| Butt et al. | Denmark, cohort | 4842 | 73 vs. 50 | 1.05 (0.89–1,23) |
| Zhang et al | China, multicenter | 4305 | 66–57 | 0.58 (0.43–0.80) |
| Saeed et al. | USA, single center | 4252 | 69 | 0.88 (0.84–0.92) |
| Grasselli et al | Italy, multicenter | 3988 | 63 | 0.98 (0.81–1.20) |
| Torres-Pena et al | Spain, multicenter | 2921 | 73 | Risk of death when statin treatment continued vs. discontinued after hospital admission: 0.67 (0.54–0.83) |
| Gupta et al | USA, multicenter | 2626 | 70 | 0.59 (0.38–0.63) |
| Lala et al | USA, single center | 2736 | 66 | 0.57 (0.47–0.69) |
| Cariou et al | France, multicenter | 2449 | 70 | 1.46 (1.08–1.95) |
| Masana et al | Spain, multicenter | 2157 | 67 | 0.60 (0.39–0.92) |
| Fan et al | China, multicenter | 2147 | 65 | 0.25 (0.07–0.93) |
| Yetmar et al | USA, single center | 1295 | 65 | 1.14 (0.64–2.03) |
| Nicholson et al | USA, single center | 1042 | 64 | 0.50 (0.27–0.93) |
| Lohia et al | USA, single center | 1014 | 65 | 0.56 (0.37–0.83) |
| Aparisi et al | Spain, multicenter | 840 | 73.5 | 0.48 (0.30–0.77) |
| Tan et al. | Singapore, single center | 717 | 46 | 0.96 (0.85–1.08) |
| Bifulco et al. | Italy, single center | 541 | 65 | 0.75 (0.26–2.17) |
| Alamdari et al. | Iran, single center | 459 | 62 | 0.27 (0.11–0.64) univariate |
| Chacko et al | USA, single center | 255 | 65 | 0.14, (0.03–0.61) |
| Soleimani et al. | Iran, single center | 254 | 66 | 0.93 (0.49–1.76) univariate |
| Song et al | USA, single center | 249 | 62 | 0.88 (0.37–2.08) |
| Daniels et al | USA, single center | 170 | 59 | 0.29 (0.11–0.71) death or intensive care unit admission |
| Krishnan et al. | USA, single center | 152 | 66 | 1.68 (0.89–3.14) univariate |
| Rodriguez Navara et al | USA, single center | 87 | 68 | 0.38 (0.18–0.77) |
| Rossi et al | Italy, single center | 71 | 72 | 0.52 (0.18–1.50) univariate |
